# Visions for our future regional electricity system: Citizen preferences in four EU countries

**DOI:** 10.1016/j.isci.2024.109269

**Published:** 2024-03-12

**Authors:** Franziska Mey, Johan Lilliestam, Ingo Wolf, Tim Tröndle

**Affiliations:** 1 Energy Transitions & Public Policy Group, Research Institute for Sustainability (RIFS) - Helmholtz Centre Potsdam, Potsdam, Germany; 2 Sustainability Transition Policy, Friedrich-Alexander University Erlangen-Nürnberg, Nürnberg, Germany; 3 Climate Policy Lab, ETH Zürich, Zürich, Switzerland

**Keywords:** Applied sciences, Electrical system, Social sciences

## Abstract

As climate targets tighten, all countries must transition toward a renewable electricity system, but conflicts about generation and infrastructure deployment impede transition progress. Although the triggers of opposition are well studied, *what people want* remains understudied. We survey citizen preferences for a renewable electricity future through a conjoint analysis among 4,103 individuals in Denmark, Portugal, Poland, and Germany. With our study we go beyond the Likert scale survey approach specifically seeking trade-offs and contextualized preferences for regional electricity system designs. We show the importance of identifying both the “least preferred” and “most preferred” solutions and highlighting the possibility of identifying very different systems with identical utility. Lastly, our research actively bridges the divide between social aspects and techno-economic modeling, promoting their integration. We show that the most preferred system design in all four countries is a predominantly regional one, based on rooftop solar, communally owned, and not relying on transmission expansion.

## Highlights

• Unearth citizen preferences for renewable electricity future in conjoint experiment
• Identified trade-offs of least and most liked options at regional level in EU
• Showed the possibility of very different systems with identical utility
• Findings pave the way for future sytem modeling by integrating societal preferences

## Graphical abstract

**Figure:**
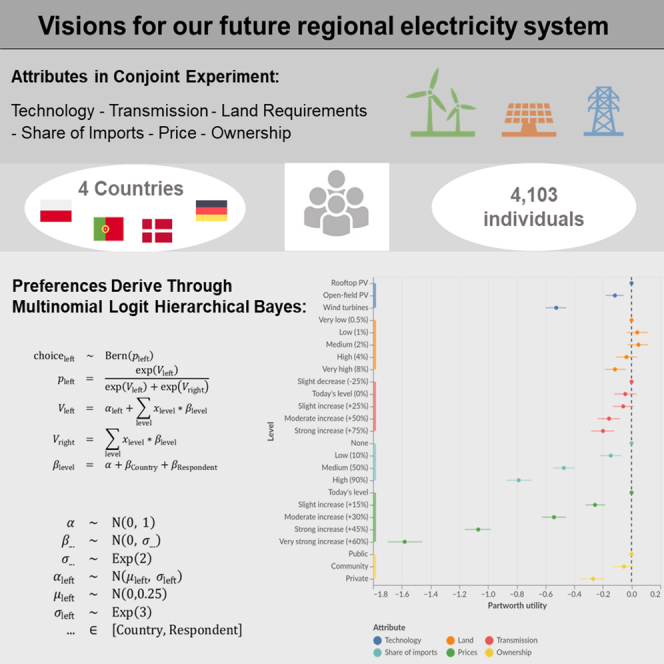

## Introduction

Multiple crises have amplified pressure to accelerate the decarbonization of the energy system. As time is waning to meet the temperature targets of the Paris Agreement,^1^ the energy crisis triggered by the Russian invasion of Ukraine has added not only political weight to immediate action for renewable energy in the 2030 target-year context but also advanced political efforts for 100% renewables by mid-century, particularly in the power sector.^2^^,^^3^

Achieving these targets will require substantial energy infrastructure development moving from a fossil-fueled power supply to a renewables-based one, dominated by wind and solar power. The expansion of renewables requires considerable land area, both for generation and infrastructure upgrades.^4^^,^^5^ This has become a contested issue particularly for local implementation, both in Europe^6^^,^^7^ and elsewhere.^8^^,^^9^ Although public polls continuously indicate high public support for the energy transition,^10^ local opposition is on the rise across Europe, resulting in delays or even failure of renewable power projects.^11^^,^^12^^,^^13^^,^^14^^,^^15^^,^^16^^,^^17^^,^^18^

Over the last three decades a large body of literature emerged, theorizing technology acceptance and opposition in relation to several socio-political, market and community factors.^19^^,^^20^^,^^21^^,^^22^^,^^23^ Empirical analyses provided insight on energy infrastructure conflicts with different emphasis on community attitudes and behavior, impacts, and governance issues.^15^^,^^16^^,^^17^^,^^24^^,^^25^ Early investigations considered objections to siting decisions through the lens of “not in my backyard” sentiments, suggesting that these objections are motivated by reasons of selfishness, ignorance, and irrationality.^26^^,^^27^ This understanding was broadly criticized as too narrow, whereas additional aspects such as place attachment, conditionalities, or lack of procedural and distributive justice emerged as drivers of opposition.^7^^,^^13^^,^^28^^,^^29^^,^^30^^,^^31^ This was confirmed in subsequent studies, showing the importance of political and economic participation.^31^^,^^32^ However, studies also conclude that process-related factors are of moderate importance, whereas directly project-related aspects, such as the location of new infrastructure or its environmental impact, strongly determine acceptance.^32^ Several recent studies question the normative connotation of conflict as something to be overcome,^14^^,^^33^ instead considering people’s meaning-making as socially embedded and co-constructed.^34^^,^^35^ More specifically, researchers suggest considering communities not as a source of opposition but as *communities of relevance*, urging future research to engage with responses to renewable energy deployment plans other than opposition, asking *what they long for*.^22^

This critical wave of research on people’s responses to renewable energy was accompanied by a shift beyond traditional methods (e.g., focus groups, interviews, and questionnaires).^36^^,^^37^^,^^38^ Multi-criteria decision analysis (MCDA), contingent valuation, and choice modeling are increasingly used to examine attitudes across different factors. Often, public acceptance is viewed as a trade-off between individual benefits and problems associated with a technology,^39^ such as expected economic and environmental impacts.^16^ Most studies using choice experiments focus on wind power, examining externalities associated with the physical attributes of wind farms, such as turbine height and setback distances, with immaterial ones like local added value (e.g., jobs) and financial benefits.^14^^,^^40^^,^^41^^,^^42^^,^^43^ For example, researchers conducted a controlled choice experiment study in Norway, finding that citizens have high acceptance for increasing renewable power production but low acceptance for additional onshore wind power.^42^ Instead, they suggest that other renewables are preferred and recommend further investigation.

Hence, there is much knowledge about drivers of opposition against renewables, but much remains unknown. First, empirical knowledge is limited to particular cases and often explores acceptance focusing on one technology (e.g., wind power), aspect (e.g., participation formats), or country.^44^^,^^45^ More importantly, there is no systematic and experimental evidence about preferences to inform political discussions about trade-offs between different power system design options, specifically considering electricity generation and distribution systems consisting of renewable energy technologies and grid infrastructure. Energy models are increasingly used to support decision-making, with high techno-economic sophistication but with weak or no representation of social aspects such as public preferences.^46^ Yet, knowing this could allow for preference-led electricity scenarios, based not on cost-optimality or avoiding what people do not want, but on what they do want. Indeed, too often people are presented ex-post with modeled scenarios and asked to react to them. In our study, we give people the choice to state their preferences *ex ante*, in a way that can be used as input to modeling. We investigate citizen preferences in several countries across multiple attributes through a conjoint experiment and survey, seeking to both increase understanding of public preferences for power system decarbonization and enable the inclusion of preferences into energy models. Hence, we make contributions on specifically two fronts. Firstly, we emphasize the importance of identifying both the “least preferred” and “most preferred” solutions and identify preferences for very different electricity design options. Secondly, we go beyond the Likert-scale survey approach and contextualized preferences for energy system designs at regional level. More specifically, we identify trade-offs between less preferred aspects and those with greater support. Ultimately, our study can be used to enhance future system modeling by integrating societal preferences in selected countries.

We show that across the four countries, electricity price, share of imports (from beyond the region that includes both national and international), and technology choice have the largest effect on citizen preferences and that it is possible to trade-off strong impacts on one factor against smaller impacts on the other factors.

## Results

There are many ways to design an electricity system based on different technologies, with transmission as the main or a minor means of flexibility provision, by producing most electricity near home or importing everything, with strongly different impacts on land use and costs.^47^^,^^48^^,^^49^ To identify citizen preferences for different types of renewable power futures, we designed a conjoint experiment along six system attributes known to affect project and policy acceptance, including technology choice and household prices (Table 1). Although we acknowledge that not all attribute combinations may provide systems that are technically feasible or efficient, we emphasize that our focus is on people’s preferences. In fact, providing technically feasible scenarios goes beyond the scope of this study and would require additional techno-economic system modeling. Instead, we asked respondents what they prefer out-of-system elements to elicit trade-offs between the least and most liked options. Ultimately this is the first step in establishing utility functions, enabling the modeling of preference-led and technically feasible scenarios.

**Table 1:** Attributes and levels for the conjoint experiment

Attributes and descriptions	Attribute levels
Technology - Main renewable power generation technology deployed in their region.	• Open-field PV • Solar PV on roofs • Wind turbines on land
Transmission - The number of overhead line masts in their region.	• Slight decrease (−25% compared with today) • Today’s level (+/− 0) • Slight increase (+25% compared with today) • Moderate increase (+50% compared with today) • Strong increase (+75% compared with today)
Land requirements - Area (excluding roofs) used for renewable power installations in their region.	• Very low (0.5% in their region) • Low (1% in their region) • Medium (2% in their region) • High (4% in their region) • Very high (8% in their region)
Share of imports - Share of electricity imported to their region	• None—all electricity comes from regional generation • Low—10% of their electricity comes from imports • Medium—50% of their electricity comes from imports • High—90% of their electricity comes from imports
Price - Electricity price development for households in their region	• Today’s level (+/− 0) • Slight increase (+15% compared with today) • Moderate increase (+30% compared with today) • Strong increase (+45% compared with today) • Very strong increase (+60% compared with today)
Ownership - The main owner of the renewable power generators in their region	• Local and regional communities—cooperatives or non-profit associations • Public sponsors—municipal utilities or municipal associations • Private utilities

We asked citizens which electricity system design they would prefer in their region, requiring them to repeatedly choose between two options consisting of different attribute level combinations, thereby revealing their relative preferences. We measure preferences through random utility theory, assuming that each option has a certain utility to each citizen. In particular, we derive partworth utilities of all attribute levels to explain the increase or decrease of utility—and therefore preferences—when adding an attribute level.

We selected four countries—Denmark, Germany, Poland, and Portugal—to reflect diversity across geographical, economic, and (energy) historical characteristics and the progress of their national energy transitions (Table S1). The experiment was introduced to the respondents using a specific framing, asking about their region as the “‘intermediary’ level,” positioned between the national and community levels. It was strategically employed to prompt individuals to consider their immediate local and regional surroundings when making decisions. Although people indeed have strong connections to their towns and cities, identity is frequently linked to larger regions. Individuals commonly consider themselves as part of regions such as Silesia (Śląsk/Poland) or Zealand (Sjælland/Denmark), in addition to their national identity. Furthermore, many people also identify themselves as Europeans, leading to descriptions like “Silesian, Polish, European,” for example. Our survey was administered to a sample of 4,189 respondents; after eliminating incomplete or unrealistic responses (e.g., <2 s response time), the final sample holds 4,103 responses, practically equally distributed across countries (total N = 4,103; Denmark N = 1,034, Germany N = 1,031, Poland N = 1,023 and Portugal N = 1,015).

This study focuses on various European countries, chosen to represent a wide range of geographical, demographic, socio-economic, and historical-cultural differences within the EU. The selection criteria encompass factors such as geographical size, population, rural population share, GDP per capita, and progress in the energy transition. Although not exhaustive, this selection aims to illustrate preferences across strongly different European Union contexts. The respondents correspond well to the properties of the population (see Table S2). We have a slight overrepresentation of rural populations, as renewable power and infrastructure tend to affect these regions and their communities more than cities.

### Price and imports are the dominant attributes

**Figure 1:**
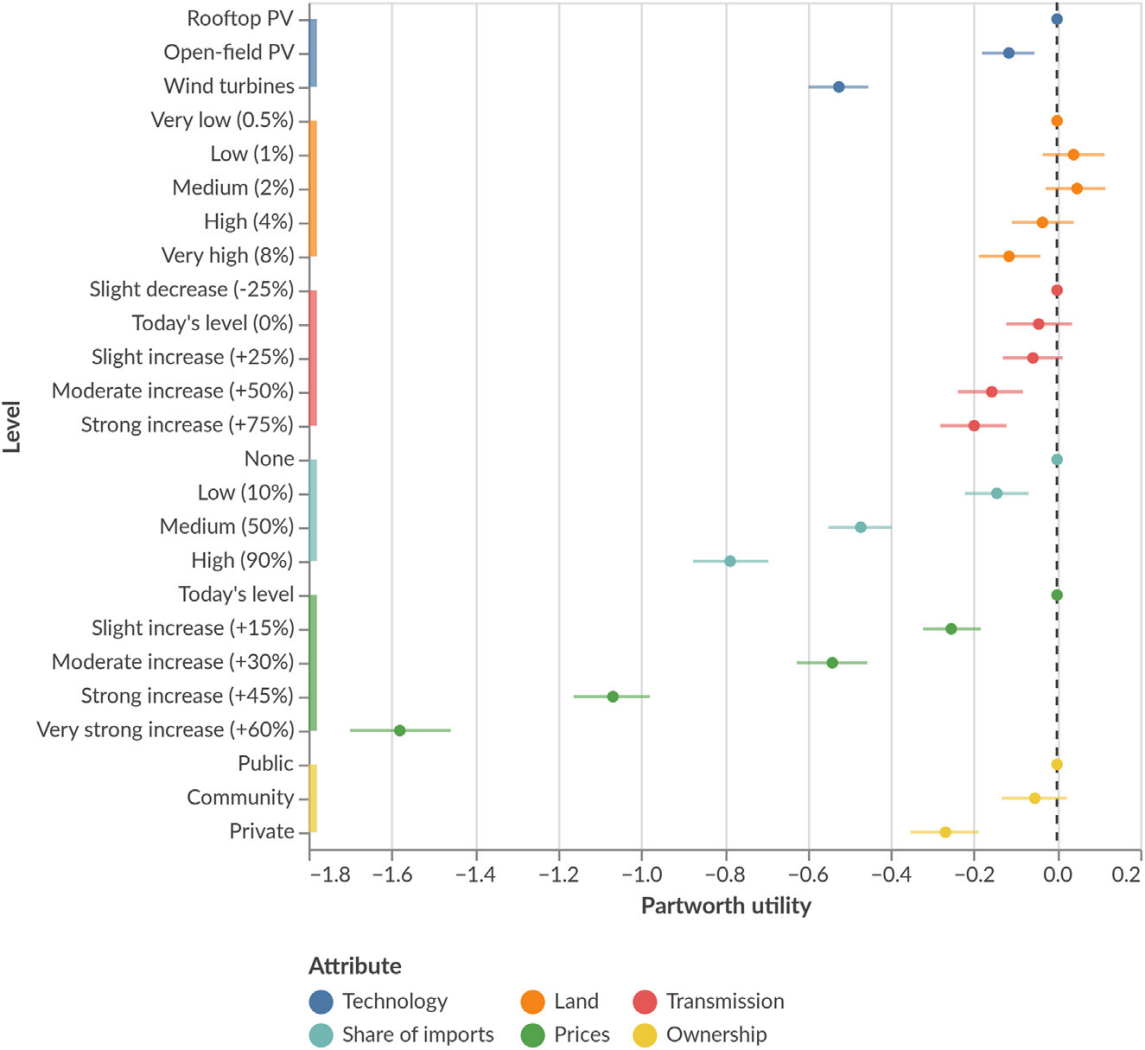
Average partworth utilities of electricity system attribute levels across all countries, showing respondents’ preferences for their respective regional electricity system Dots represent the expected values (means of plausible values), and the bars show estimation uncertainty (all plausible values within the 94% highest density interval). Higher values indicate more preferred attribute levels. Total utility of system designs can be derived by summing partworth utilities across attributes. N = 4,103 respondents and N = 32,824 choices.

The reactions across attributes vary strongly (Figure 1) across the specific system design elements. The dominant technology, import share, and especially electricity price attributes show strong effects, indicating that these attributes are central to citizen preferences, whereas transmission expansion, land use, and ownership show only small differences across attribute levels, suggesting smaller importance. The import share attribute shows strong effects, with low or no imports being clearly more preferred than higher imports. Our survey results confirm this finding (see Figure S1), as most respondents support or strongly support the statement that their electricity should exclusively be produced domestically. Note that the survey was conducted just before the Russian invasion of Ukraine, reflecting attitudes under the “old normal,” with possibly stronger views on the necessity of energy import independence today.

The electricity price attribute triggers the largest effects, with reactions to increasing prices being more than twice as strong as reactions to increasing shares of imports. The survey results support this: respondents practically unanimously agree or strongly agree that the power system must be cost-effective; we find similarly high agreement with the statement that the power future must be socially just (see Figure S2). Our results show that although citizens strongly prefer domestic electricity, they very strongly prefer low electricity prices.

Finally, the strong response on technology choice is consistent with many other studies^50^^,^^51^^,^^52^: respondents prefer PV, and especially rooftop PV, over wind power as the regionally dominant technology. Nevertheless, the technology attribute reaction is smaller than the import and price attribute reactions, indicating that citizens may be willing to accept wind power if it helps reduce imports or prices.

### Preference trade-offs

Because the preferences (Figure 1) differ strongly between attributes and levels, it is possible to identify the most and least preferred future system designs. An average respondent prefers a (rooftop) PV-based system with low prices, local ownership, and regional generation, with only little transmission. The least preferred system is wind-dominated, with much import and transmission, high prices, and regional land use, owned by private companies. Hence, we find a clear preference for decentralized electricity futures (Table 2a, left scenario), if such futures are achievable without sharply rising prices and a relative rejection of centralized systems (Table 2a, right scenario).

**Table 2:** Contrasting scenarios of regional electricity designs based on the average partworth utility per attribute level

**Scenarios: Most distinct**^a^					

Technology	*Rooftop solar*	0	Technology	*Wind*	-0.53
Land	*Medium*	0.05	Land	*Very high*	-0.12
Transmission	*Slight decrease*	0	Transmission	*Strong increase*	-0.20
Imports	*None*	0	Imports	*High*	-0.79
Price	*Today's level*	0	Price	*Very strong increase*	-1.58
Ownership	*Public*	0	Ownership	*Private*	-0.27
**Utility**		**0.05**	**Utility**		**-3.47**

**Scenarios: same utility central & decentral**^b^					

Technology	*Wind*	-0.53	Technology	*Rooftop solar*	0
Land	*Medium*	0.05	Land	*Low*	0
Transmission	*Moderate increase*	-0.16	Transmission	*Slight increase*	-0.06
Imports	*High*	-0.79	Imports	*Low*	-0.15
Price	*Today's level*	0	Price	*Strong increase*	-1.07
Ownership	*Public*	0	Ownership	*Community*	-0.05
**Utility**		**-1.42**	**Utility**		**-1.32**

**Scenarios: wind vs. rooftop solar**^c^					

Technology	*Wind*	-0.53	Technology	*Rooftop solar*	0
Land	*Medium*	0.05	Land	*Low*	0.04
Transmission	*Moderate increase*	-0.16	Transmission	*Moderate increase*	-0.16
Imports	*Low*	-0.15	Imports	*Medium*	-0.47
Price	*Slight increase*	-0.26	Price	*Moderate increase*	-0.54
Ownership	*Private*	-0.27	Ownership	*Community*	-0.05
**Utility**		**-1.30**	**Utility**		**-1.18**

a most and least preferred designs.

b (practically) same-utility but highly different attribute combinations.

c illustration of a wind-based system design with the same utility as a PV-based one.

However, when designing the future power system, public preferences are merely one of several factors to consider, and possibly technical, geographical, or political reasons require some relatively undesirable option (e.g., substantial imports, large wind power shares): it is not necessarily possible to choose the most preferred future. By considering public preferences as utility, it is possible to explicitly include them in energy system models alongside other factors to find feasible and preferred system designs, and as preferences differ strongly across attributes, this is important to generate low-resistance scenarios. The differences across the attributes enable trading off preferred against less preferred solutions—a mix-and-match of specific elements to generate highly disparate options with similar total utility. For example, options (c, left) and (c, right) in Table 2 have (almost) the same utility, but whereas (c, left) holds many attributes of a wind-dominated centralized system, option (c, right) is more decentralized. Among these options, technical feasibility would need to be explored, but previous research has shown that very many and very different system designs are technically feasible, both nationally and in a European view, at similar costs.^47^^,^^48^^,^^53^ There is thus good reason to expect that many attribute combinations would lend themselves to designing a technically plausible electricity future.

This mix-and-match approach can also be made actively to “compensate” for a non-preferred option that for technical or political reasons is still necessary, allowing for the construction of similarly preferable but highly different scenarios. For example, our results show that wind power is less preferred than PV, confirming the observed opposition against wind power projects across Europe,^13^^,^^14^^,^^15^^,^^16^^,^^28^^,^^54^ but this relative rejection of wind power may be compensated through the relative preference of other attributes. Combining the less preferred wind power with more preferable attributes, such as low imports and prices while only moderately relying on transmission, may generate a similar utility as a rooftop PV-centered approach if that comes with somewhat higher prices and imports (Table 2).

Although price is the largest lever, price is also an outcome, directly influenced by (transition) policy decisions (technology mix, transmission reliance, etc.). However, a large share of household prices is determined by political instruments, like taxes, levies, and fees, which can be influenced by decision-makers.

### Similar preferences in all four countries

**Figure 2:**
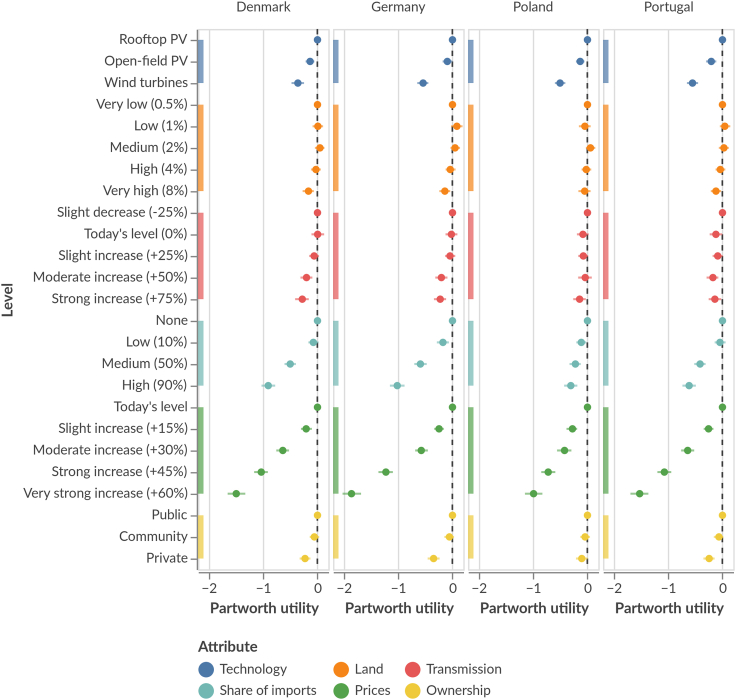
Average partworth utilities of electricity system attribute levels within each country: Denmark, Germany, Poland, and Portugal Dots represent the expected values (means of plausible values), and the bars show estimation uncertainty (all plausible values within the 94% highest density interval). Higher values indicate more preferred attribute levels. Total utility of system designs can be derived by summing partworth utilities across attributes. N = 4,103 respondents and N = 32,824 choices.

When investigating the preferences for each country individually (Figure 2), we find a similar picture as for the aggregate (Figure 1): prices, import share, and dominant technology trigger the strongest divergence across the explored levels, with the other attributes showing less variation. Germans and Danes are closely aligned in their preferences, possibly indicating similarly far-progressed transitions; Poland and Portugal show weaker reactions to imports, and Poland specifically differs regarding price preferences. This may be explained by the relatively low electricity prices in Poland (€0.15/kWh in 2021^55^; Germany: €0.32/kWh in 2021^56^) and resulting political salience of keeping prices lower than in neighboring countries. Despite these differences, the observed cross-country similarity suggests that a European-level preference-led approach to designing a renewable power system would work: as preferences are similar, acceptance-increasing strategies would be similar too. However, as preferences are dynamic and subject to change over time, it would be ideal to conduct surveys regularly, especially if we aim to provide reliable acceptance and preference data to inform modeling.

### Large range of opinions: No option to please all

**Figure 3:**
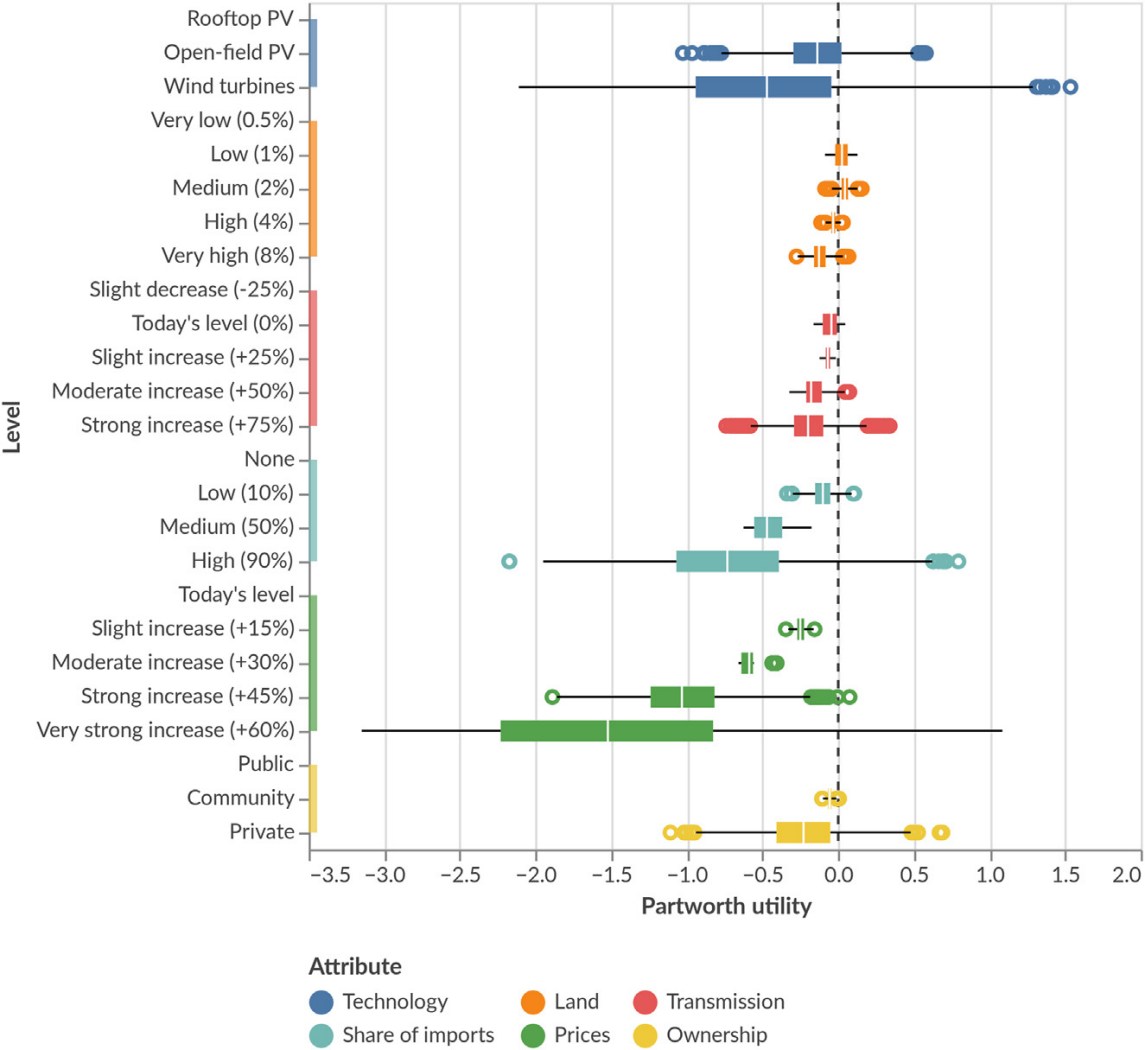
Respondent-level partworth utilities of electricity system attribute levels For each respondent, we use the mean across all plausible values. Boxes range from the 1st to the 3rd quartile across respondents. Whiskers of the boxes range maximum 1.5 times the interquartile range. Respondents outside this range are displayed as points. Higher values indicate more preferred attribute levels. Total utility of system designs can be derived by summing partworth utilities across attributes. N = 4,103 respondents.

Despite the clear average preferences (all cases and country-specific), our results also show that individual preferences differ strongly, particularly regarding the most salient attributes (price, imports, technology) (Figure 3). Indeed, there are some strong outliers on either side: for example, although most respondents do not prefer wind power, some individuals show strong support. Similarly, private ownership has several strong individual preferences on both margins. By contrast, individual preferences in land, transmission infrastructure, and community ownership are more aligned with smaller differences among individual preferences.

Looking for explanations, we clustered respondent-level covariates into geographical (urban and rural), gender, income, and education levels. However, the impact was marginal and did not explain the large variation across respondents (see STAR Methods: data analysis and Figure S6).

This means that even an electricity future tailored to the average preferences or the most preferred scenario (Figure 1; Table 2) may still trigger opposition from individuals with diverging preferences: it is not possible to generate electricity futures that cater to *all* individuals’ needs, although it is possible to identify futures preferred by most.

## Discussion

We explored citizen preferences for the design of future regional renewable electricity systems in Denmark, Germany, Poland, and Portugal and found that price, imports, and technology choices are the key drivers of citizen preferences. If citizens were to decide, the future renewable power system would be decentralized, based on supply with high shares of rooftop solar, communally owned, not relying strongly on transmission and leading to low household prices. Across all four countries, citizens prefer such a decentralized system over a more centralized, wind power- and transmission-heavy system relying on imports. These findings support previous research findings.^57^^,^^58^

However, our approach sets us apart from other large-scale studies,^59^^,^^60^^,^^61^^,^^62^^,^^63^ such as the Eurobarometer^64^ or the Sustainability Barometer,^37^ as we refrain from asking direct questions. Instead, we conducted a conjoint analysis to unearth preferences, rather than gathering Likert-scale responses. This approach is novel and enhances the robustness of our results, as there is little knowledge about trade-offs resulting from preference choices yet. It acknowledges that people may struggle to provide reliable answers to direct queries regarding a future system they are not familiar with. However, they can assess a system design, even if it is described in somewhat abstract terms, and express whether they prefer it over another design, hence elucidating trade-offs that will otherwise be missed.

The electricity price dominates the preferences, showing that keeping prices moderate is essential to satisfy citizen preferences, as too high prices may override preference gains made through other attributes. Nevertheless, our findings imply that it may be possible to trade-off less preferred aspects against such with greater support, such as accepting a wind- and import-centered system if these options help keep prices low. The preferences for electricity system designs provide important information to use alongside other factors, such as technical, geographical or political factors, when exploring and designing the renewable power system of the future. Our findings suggest that the analytical tools at hand in system planning, particularly energy system modeling, should emphasize more and better include preferences and social factors, as Süsser et al.^45^ have rightfully pointed out. Although this holds some truth, it is important to acknowledge that people do place significant emphasis on costs, which models extensively explore and scrutinize. However, we also reveal the importance of considering further, not techno-economic questions such as ownership or import dependence, as these co-decide the overall preferences. First, efforts have been made to include softer aspects in energy system modeling, such as minimizing effects on scenic landscapes,^59^ but such techno-economic-societal modeling approaches are still in their infancy. The data we generated here lend themselves to empirically based, preference-led, or -optimized modeling, and we call upon the community to keep pushing agendas to better reflect social and political preferences in energy modeling.

The unified assessment of preferences allows for the integration into highly resolved, bottom-up energy system models, so that social preferences can be assessed alongside other aspects like technical feasibility, cost, or land requirements. As our study provides a quantification not only of average preferences but also of their uncertainty and variability across contexts and individuals, they allow for including social aspects within integrated models. The structure of our data, which also allows for lower-than-national resolved analysis, enables application to various contexts and may also be at least roughly applicable to similar regional contexts in other (European) countries; for higher precision, the experiment would need to be repeated in other countries and over time, to cover both the breadth and evolution of preferences. Our experiment can be repeated without large efforts, as both the survey and the evaluation framework are public.

The integration of our results into energy system models could be performed in a soft- or hard-linked manner. In a soft-linked approach, our results can be applied to existing energy system model results within a post-processing step. The main challenge in this approach is mapping between the different data models of energy systems, as there will be a structural break between the model we use in this study and the one within the energy system model. Data mapping is also required in hard-linked approaches in which our results are embedded in the energy system model. This approach allows for a more extensive analysis of preferences of energy system designs. For example, it may allow the finding of technically feasible system designs with maximal average utility or with minimal utility variability within regions and therefore low conflict potential. Such optimizations are common in energy system models but usually are performed to minimize monetary costs in a linear way. As our results are not linear, the integration would require either a linearization of the results or the introduction of non-linearity to the energy system model.

### Limitations of the study

Our findings come with some caveats. Firstly, we generated the data in early 2022, before the Russian invasion of Ukraine, and hence in a European energy policy context that does not exist anymore and may never return. Given the energy price shock of 2022 and prevailing concerns about energy supply security, the importance of the electricity price and import attributes has likely increased today compared with our data. We call upon continued research on this, including with our questionnaire (note to Editor and Reviewers: link to questionnaire and raw data published on Zenodo will be available when published), to explore how energy transition preferences evolve.

Secondly, although previous research suggests that power systems can be designed in vastly different ways but still experience similar costs, not every combination of technologies, transmission, land use, and imports may be technically feasible, especially not at every cost level. This further supports our call for preference-led or -optimized energy system modeling, to better identify the full decision space for preference satisfaction and impacts on cost and other factors. This also refers to the political environment for which not only matching attributes to current preferences but also *creating* the dominant framing of an energy policy strategy may be important. For example, our results show that it is possible to trade-off 50% electricity imports (−0.47 partworth utility) if it avoids a 30% increase in prices (−0.54), considering all other things are equal. Hence, labels such as “freedom energy”^65^ for solar and wind energy may indeed help leverage such effects and support public acceptance, both for the energy transition and for shorter-term policies, including during hardships and price increases as Europe rids itself of Russian energy imports. Similarly, efforts to include citizens not only pro forma in “participation processes” but also in letting them invest in the new assets near their homes may increase acceptance of, for example, wind-heavy system development plans that may otherwise encounter resistance.^14^

Yet, these trade-offs apply specifically to preferences. In a techno-economic setting, as opposed to our preference elicitation setting, the attributes may not be entirely independent (e.g., PV and wind power have different land needs, imports may generally reduce regional electricity prices, etc.). Whether or not such a trade-off or attribute combination is technically feasible—e.g., whether a specific region can indeed trade lower imports for higher prices—is beyond the scope of this study and must be explored with an energy system model. Although this study does not investigate technical feasible scenarios, instead explores combinations of attributes that citizens prefer or not, continuous research can support preference-led energy modeling considering technical feasible options and future support preference-led energy system modeling in continued research.

Thirdly, in our experiment we only asked for specific design choice preferences and did not consider preferences about technology mixes. In a real, materialized renewable power system, one technology may be dominant but will not be the sole technology; future surveys may thus seek to explore preferred energy mixes, going further than investigating preferences for single dominant technologies.

Ongoing energy policy efforts in Europe are well aligned with the citizen preferences we identify here. Notably, the EU’s *Clean Energy for All* package sent a strong signal to Member States to strengthen decentralized and participatory structures in their national energy transition policies, acknowledging citizens as important actors in the energy market and outlining governance principles for energy communities.^66^ At the same time, the *REPowerEU* plan for meeting the updated renewable energy target (45% by 2030) pushes for an accelerated expansion of large-scale renewables.^2^^,^^67^ Such two-pronged approaches, aiming at both centralized and decentralized expansion, may appear inconsistent, but they may also be a “clumsy solution,”^68^ offering solutions that cater to the preferences of very different groups, thus building acceptance among diverse societal actors and ultimately decreasing conflict.

## STAR★Methods

### Key resources table

**Table:** 

REAGENT or RESOURCE	SOURCE	IDENTIFIER
**Deposited data**		
Raw survey data	This paper	https://zenodo.org/doi/10.5281/zenodo.10463073
Result data: pre-processed survey data and inference results of our statistical models	This paper	https://zenodo.org/doi/10.5281/zenodo.10463113
Workflow code to reproduce results	This paper	https://zenodo.org/doi/10.5281/zenodo.10463119

### Resource availability

#### Lead contact

Further information and requests for resources should be directed to and will be fulfilled by the lead contact, Franziska Mey (franziska.mey@rifs-potsdam.de).

#### Materials availability

This study did not generate unique reagents.

#### Data and code availability

Original data have been deposited at Zenodo (https://zenodo.org/) and are publicly available as of the date of publication. Accession number is listed in the key resources table.

The code for analysis has also been deposited at Zenodo and is publicly available under the accession number listed in the key resources table.

Any additional information required to reanalyze the data reported in this paper is available from the lead contact upon request.

### Method details

#### Experimental design

To assess citizen preferences for renewable electricity supply in their region, we conducted a choice experiment. Choice experiments are a widely used method to assess stated preferences in social and political science.^69^^,^^70^ In our experiment, respondents are repeatedly presented with a choice between two hypothetical designs of the electricity supply system in their region. Each option consists of six attributes describing the electricity supply: dominant technology, land requirements, level of electricity imports into the region, household electricity prices, overhead transmission capacity expansion, and ownership of the assets (see Table 1 for all attributes and attribute levels; see Figure S3). Each participant was presented with eight such choices from which we identified the relative importance of the attributes and their 25 levels. We randomised the combinations of attribute levels (fully randomised design, see Figure S3) and the order in which attributes are presented (across respondents, but not within respondent). The randomisation also led to combinations that might appear technically infeasible. However, our main priority was to identify preferences, not technical feasibility. The assessment of whether combinations are technically viable necessitates examination with a (technical, techno-economic) energy system model. Consequently, the exploration of technical feasibility succeeds the elicitation of preferences. Our approach avoids generating technically feasible or optimal scenarios for subsequent evaluation; rather, we centre on the identification of preferences as a first step, and these preferences may subsequently be used in preference-driven system modelling.

The technology choices were based on widely distributed and well-known options that play the dominant role in the energy transition. We excluded technology options such as storage, yet not because we think it’s not necessary for technical feasibility but rather because we believe their impact on preferences is negligible or minimal. In contrast, the attribute of electricity price is a significant factor in the public discourse, and we focused solely on rising prices. This approach acknowledges that while generation costs themselves may decrease in the future, grid and balancing costs – and hence final electricity prices - are likely to rise. The regional focus in pricing is also pertinent given the ongoing political discussions about pricing reforms in several Member States.^71^^,^^72^^,^^73^ Intra-national price zones, rendering more local pricing are likely reaction to the more local localisation of renewables, compared to fossil sources. So, if decisions on system design are regional, then the price impacts are or are likely to become regional too.

The choice experiment was set up through conjointly.com and distributed with support from polling agencies in each country. Each respondent was presented with eight consecutive pairs of hypothetical regional system designs for a future fully renewable electricity supply with the task of selecting between two options. The display of attribute levels was enhanced with small pictograms for enhance the understanding (see Figure S7).

#### Sampling

We conducted our choice experiment as an online survey between 24 January and 8 February 2022 using the platform conjointly.com. Participants were recruited from a commercial volunteer online access panel administrated by Respondi AG and partners in the four countries. From these access panel, we drew a proportional quota sample (Germany n=1,031, Poland n=1,023, Denmark n=1,034 and Portugal n=1,015) using gender and residential location as quota variables approximating the stratification of populations on these indicators in the respective countries (see Table S2). The sample in the four countries holds in total N=4,103 and the median survey duration was 7.0 minutes.

#### Country selection

This study focuses on European countries due to the shared EU energy policy framework while maintaining different paces in their energy transition progress (see also Note S1). The specific countries were chosen to reflect the high diversity of geographical, demographic, and socio-economic as well as historic-cultural differences across Europe. The selection criteria include the geographical size see Table S1 a), the size of population (b) and share of rural population (c); economic position and living standards represented in GDP per capita (d); and the progress in the energy transition represented by the share of renewables in final electricity demand (e), gross final energy consumption (f) and wind energy capacity (g and h). The country selection is not representative of all EU countries but is meant to be illustrative of preferences in different places and contexts of the European Union.

#### Data analysis

We derive preferences from recorded choices using multinomial logit hierarchical bayes, a method commonly used for choice experiments.^74^ It is based on the random utility theory and assumes that each option has a distinct utility to each respondent and that respondents choose the option with higher utility. As we did not measure utility but choices, utility is a latent variable that is estimated by the model. Apart from total utility of an option, we also estimate partworth utilities of all attribute levels. Partworth utilities are the main constituents of total utility, and they explain the increase or decrease of utility arising from adding an attribute level to the option. Therefore, partworth utilities are conceptually comparable to Average Marginal Component Effects (AMCE), even if the scale is different. The full model is shown in Equation 1.

Given that we have exactly two options per task, we model choices as a Bernoulli variable (first line in Equation 1. The probability of the Bernoulli variable depends on the deterministic parts of the utilities of both options (left option and right option, second line in Equation 1. Utilities (V) are linear combinations of the partworth utilities of each level that is included in an option (third and fourth line in Equation 1. For example, if the left option showed an electricity supply based mainly on publicly owned wind turbines within the region, utility is the sum of the partworth utilities of the attribute levels of “public utility” and “wind turbine”, and the four other attribute levels included in the option. Being a logistic model, it’s important to note that the attribute levels are not independent; rather, they interact. To capture the possibility that respondents preferred the left or right option irrespective of the shown attribute levels, we add an intercept term to the left utility (third line in Equation 1. The intercept terms are very small or zero (see Figures S4 and S5), showing that there is no impactful bias towards left or right options. Finally, the partworth utility of each level is the sum of level-specific intercept and varying (random) effects. In the base model, we add a varying effect for country (N=4) and a varying effect for each respondent (N=4,103).

(Equation 1) $$\begin{array}{c} \begin{array}{cc} {choice}_{left}\;∼ & Bern\left(p_{left}\right)\\ \;{\;p}_{left}= & \frac{\exp\left(V_{left}\right)}{\exp\left(V_{left}\right)+\exp\left(V_{right}\right)}\\ \;V_{left}= & \alpha_{left}+\sum_{level}x_{level}*\beta_{level} \end{array}\\ \begin{array}{cc} {\;V}_{right}= & \sum_{level}x_{level}*\beta_{level}\\ \;\beta_{level}= & \alpha +\beta_{Country}+\beta_{Respondent} \end{array} \end{array}$$

Being a Bayesian model, we add prior probabilities for all parameters (Equation 2). We use weakly informative priors to avoid unrealistically large parameter values. Given that utilities are defined on the logit scale in this model, a utility value of 4 or -4 means that an option is chosen or rejected with a probability larger than 98% (when the utility of the other is 0). Therefore, we deem partworth utilities with absolute value larger than four unrealistic and tune the prior probabilities accordingly. We model varying effects of country and respondent with mean zero (second line in Equation 2) as the models includes a separate intercept term per partworth utility (first line in Equation 2, bottom line in Equation 1. We do not model covariances between attribute levels as the additional computational complexity is restrictive.

We implement our probabilistic model using PyMC.^75^ Code and data to reproduce our analysis are publicly available [note to Editor and reviewers: after publication of the article]. We sample the posterior distribution of all model parameters using a NUTS sampler. We run four independent chains of the Markov chain Monte Carlo to check for convergence. Each chain iterates a total of 4,000 times of which 2,000 iterations are tuning steps which we discard. The chains converge to the posterior distributions (see Table S3).

In addition to the base model, we implement a model that includes respondent-level covariates as

(Equation 2) $$\begin{array}{ccc} \alpha & ∼ & N\left(0,1\right)\;\\ \beta_{\ldots } & ∼ & N\left(0,\sigma_{\ldots }\right)\;\\ \sigma_{\ldots } & ∼ & \mathrm{Exp}\left(2\right)\;\\ \alpha_{\text{left}} & ∼ & N\left(\mu_{left},\sigma_{left}\right)\;\\ \mu_{\text{left}} & ∼ & N\left(0,0.25\right)\;\\ \sigma_{\text{left}} & ∼ & \mathrm{Exp}\left(3\right)\;\\ \ldots & \in & \left[Country,Respondent\right] \end{array}$$

varying effects. We add age (N=7), gender (N=3), education (N=7), and area (urban/rural, N=2) varying effects to better estimate the potential bias introduced through non-random sampling. In this covariate model, all additional varying effects are added to the partworth utilities of each attribute level (Equation 3). We find that the impact of these respondent-level covariates is small and with it the bias introduced through non-random sampling see Figure S6). Therefore, we exclude these covariates in the base model.

While the data are complete for the experimental variables and no further treatment is necessary for the base model, there are missing values for the covariates, which require further treatment to be used in the covariate model (Equation 3). There are missing values in the age, education, and area covariates, for which 17, 40, and 54 respondents respectively did not state valid values (0.4%, 1.0%, 1.3%). We use single, multivariate data imputation^76^ to fill in the missing values. The out-of-bag error of the imputation is 0.01, 0.32, and 0.35, respectively for the age, education, and area covariates.

(Equation 3) $$\beta_{level}=\alpha +\beta_{Country}+\beta_{Age}+\beta_{Gender}+\beta_{Education}+\beta_{Area}+\beta_{Respondent}$$

## Supplemental information

Document S1. Figures S1–S7, Tables S1–S3, and Note S1
