# Supplementary material for: Visions for our future regional electricity system: Citizen preferences in four EU countries
Source: iScience. 2024 Mar 12;27(4):109269. doi: 10.1016/j.isci.2024.109269 (PMC11074965; doi:10.1016/j.isci.2024.109269)
Supplement: Document S1. Figures S1–S7, Tables S1–S3, and Note S1 [file mmc1.pdf]

iScience, Volume *27*

Supplemental information

Visions for our future regional electricity system: Citizen preferences in four EU countries

Franziska Mey, Johan Lilliestam, Ingo Wolf, and Tim Tröndle

Supplemental information

Figure S1: Survey responses on electricity imports, related to Results - Price and imports are the dominant attributes, related to Figure 1.

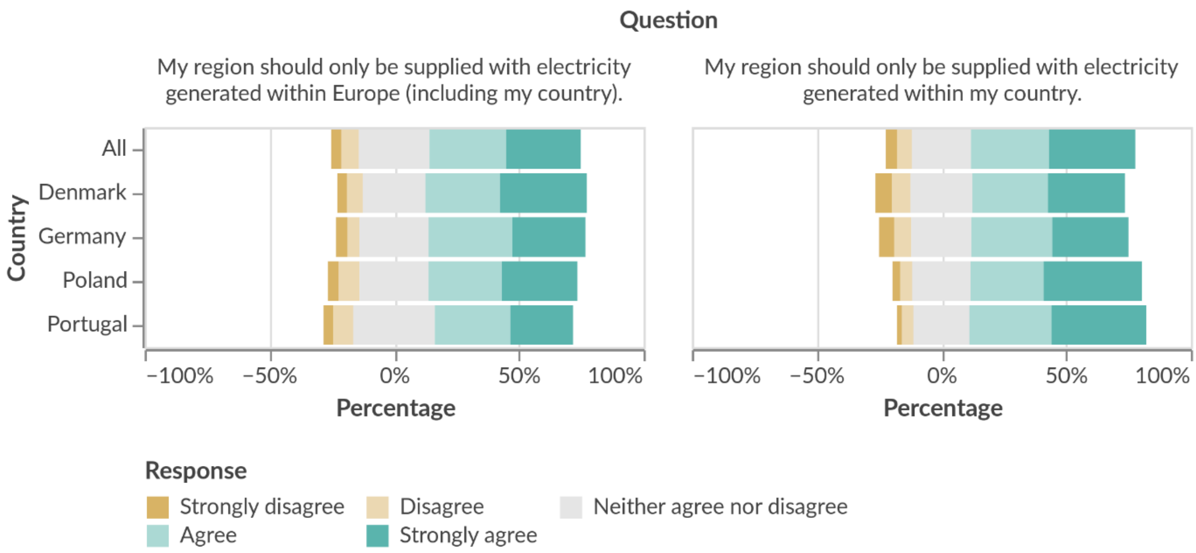

Figure S2: Survey responses on justice, related to Results - Price and imports are the dominant attributes, related to Figure 1.

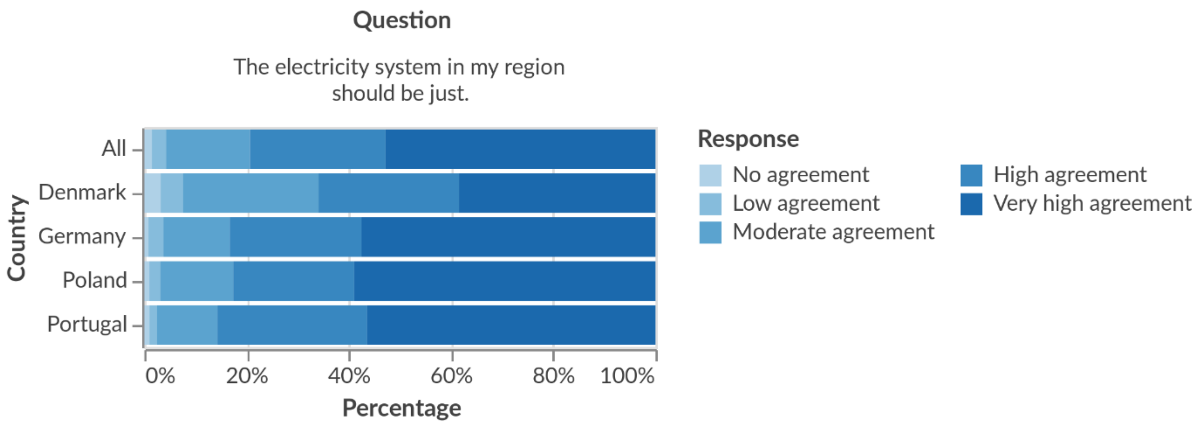

Figure S3: Probability of each pair of attribute levels to appear within the same profile. Probability is larger within attributes with fewer levels, related to Table 1 and Star Methods - Experimental Design.

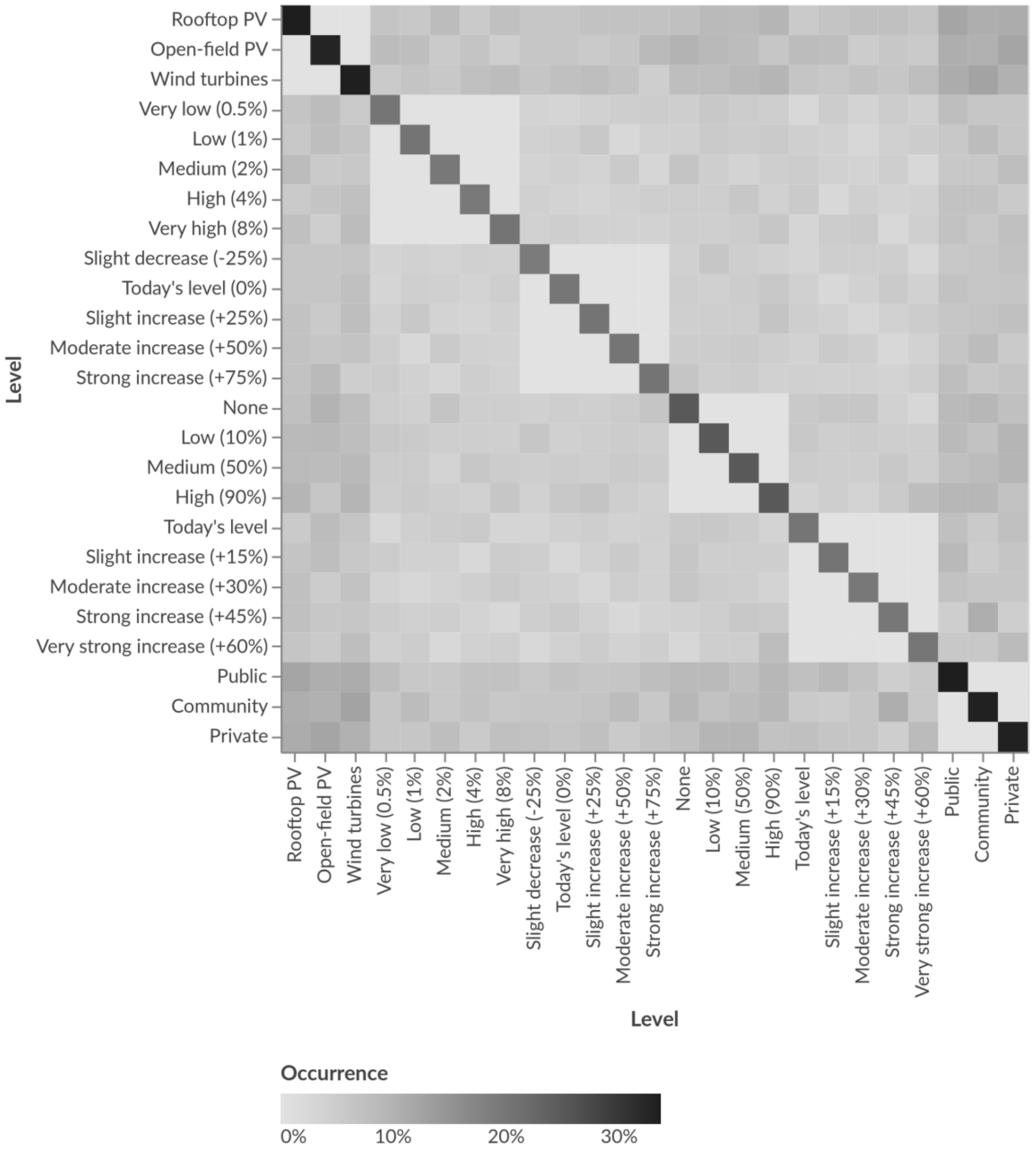

Figure S4: Posterior distributions of the varying effect of displaying options on the left-hand side compared with displaying them on the right-hand side, related to Star Method – Data analysis.

A) Description of panel A: Expected value and uncertainty of the effect for each respondent. The dark line shows the expected value (the mean of the posterior distribution). The two shaded areas show the uncertainty (60% and 94% highest density intervals). Respondents are sorted by their expected value.

B) Description of panel B: Posterior distribution of the population-level average.

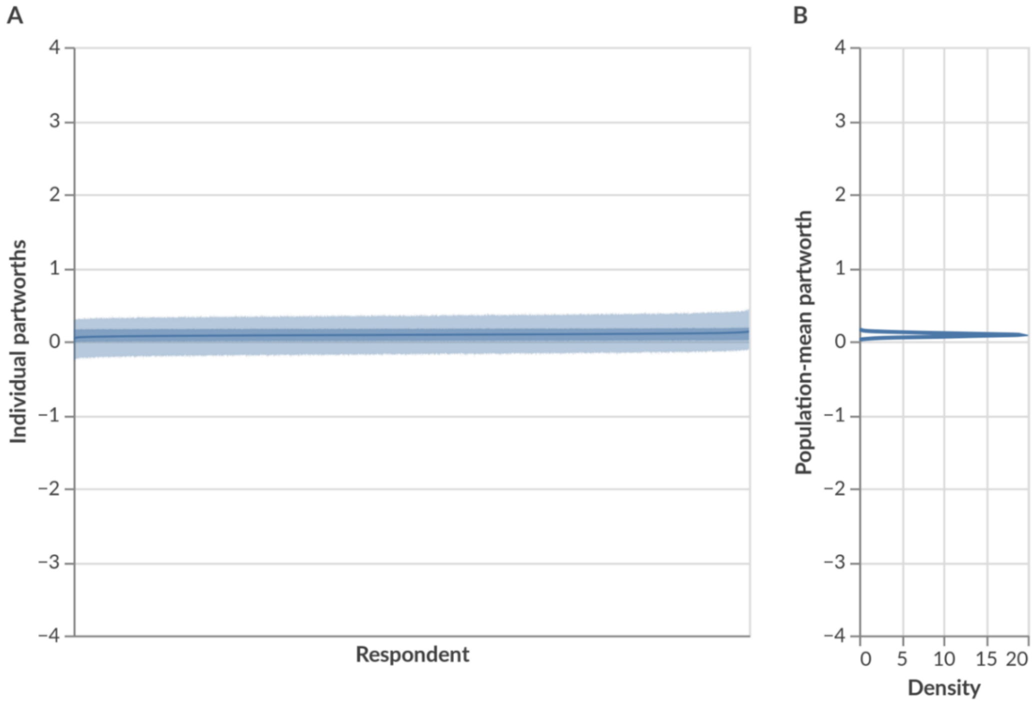

Figure S5: Posterior distributions of the varying effect of wind turbines, related to Star Method – Data analysis.

A) Description of panel A: Expected value and uncertainty of the effect for each respondent. The dark line shows the expected value (the mean of the posterior distribution). The two shaded areas show the uncertainty (60% and 94% highest density intervals). Respondents are sorted by their expected value.

B) Description of panel B: Posterior distribution of the population-level average.

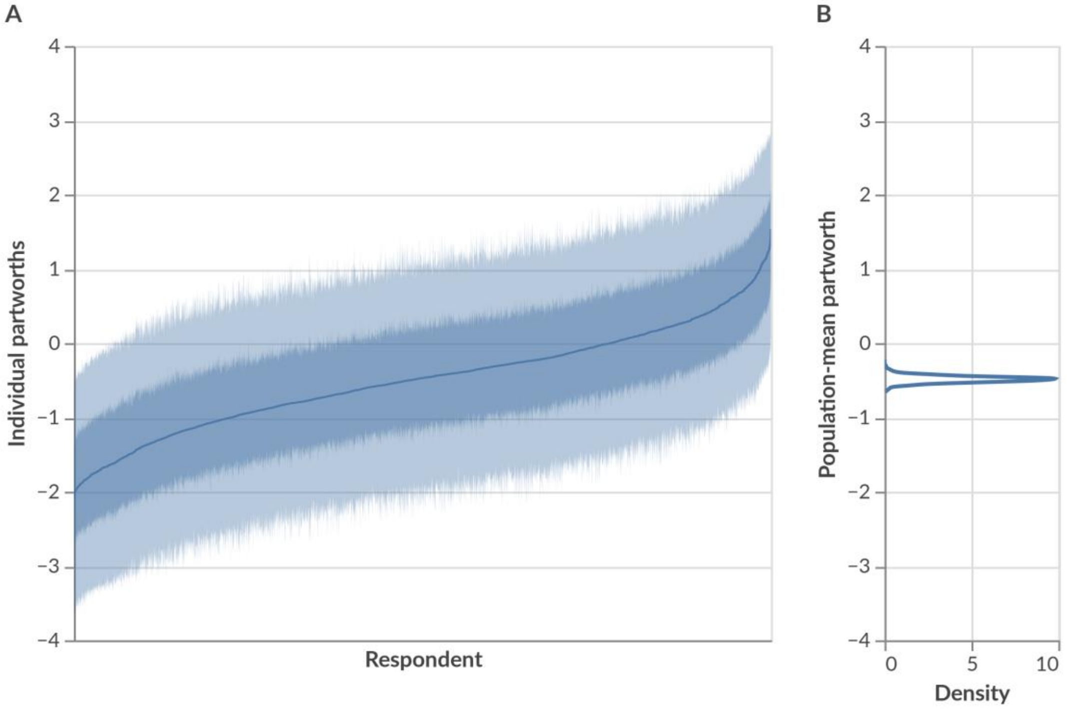

Figure S6: Posterior distributions of the largest varying covariate effect across subgroups, related to Star Method – Data analysis, Figure 3

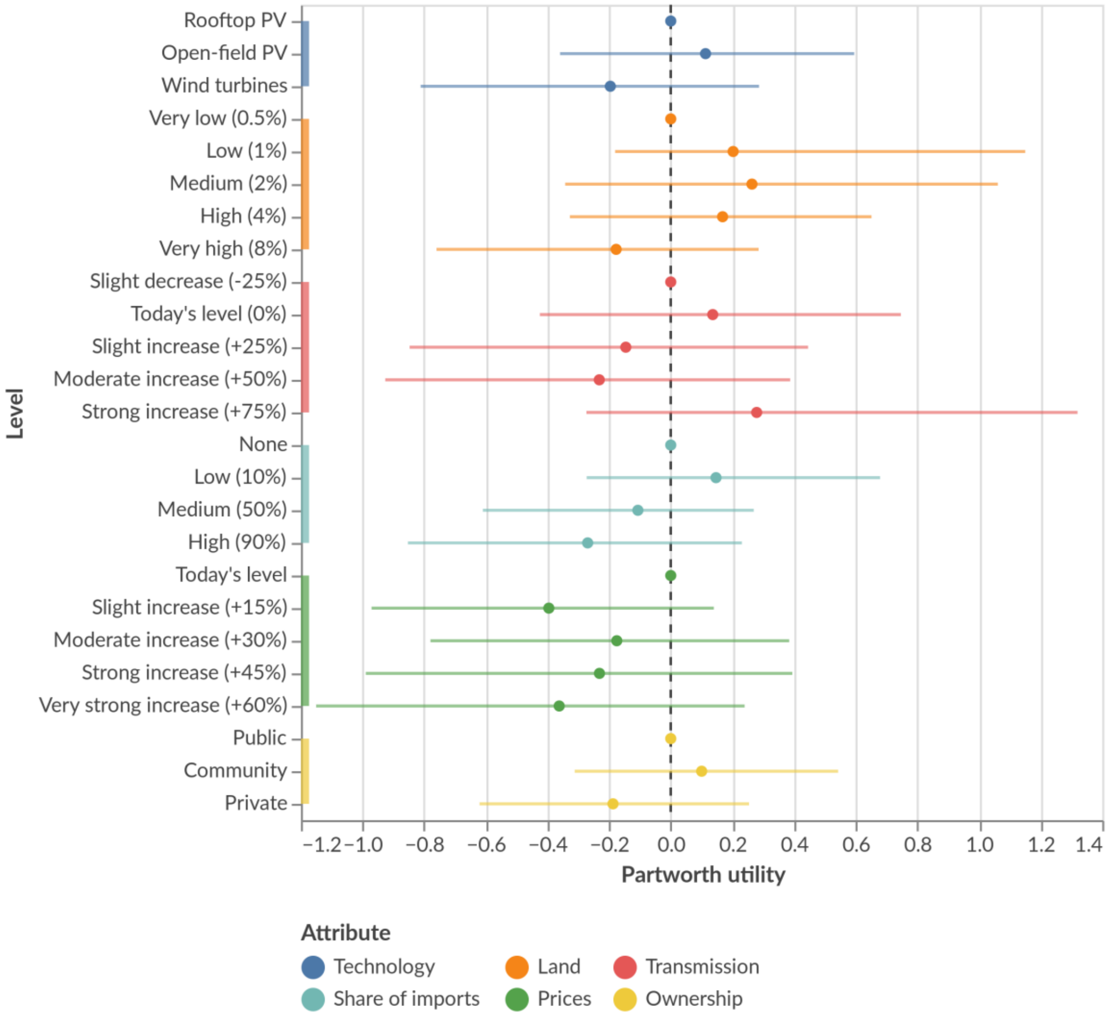

Figure S7: Example of the choice display, related to Table 1 and Star Method - Experimental design.

Qual das duas opções para um **futuro possívelsistema eléctrico** que prefere para a **sua região**?

Definimos uma região como a sua morada num raio de cerca de 30 km, incluindo as áreas naturais adjacentes.

Todas as opções são concebidas para satisfazer as suas necessidades regionais de electricidade.

	**Opção 1**	**Opção 2**
**Tecnologia**<br><br>Centrais de energias renováveis que são principalmente utilizadas para produzir eletricidade na sua região.	Turbinas eólicas em terra	Sistemas fotovoltaicos em telhados
**Propriedade das instalações de produção**<br><br>A quem pertencem as centrais de energias renováveis?	Patrocinadores públicos - serviços públicos municipais ou associações municipais.	Comunidades locais e regionais - cooperativas ou associações sem fins lucrativos.
**Área necessária**<br><br>Área (sem áreas de telhado) utilizada para instalações de energias renováveis na sua região. Para comparação, os assenta-mentos em Portgual ocupam 8%.	↑↑ Muito alta (8% na sua região)	↑ Muita (4% na sua região)
**Quantidade de importações de eletrici-dade**<br><br>Quota de eletricidade produzida fora da sua região.	Nenhuma – a sua eletricidade provém de instalações regionais.	↑ Alta – 90% da sua eletricidade provém de importações.
**Remoção dos postes de linhas áreas**<br><br>Reduzir ou expandir o número de postes de linhas aéreas na sua região.	↘ Redução ligeira (-25% em comparação com a atualidade)	↗ Aumento moderado (+50% em comparação com a atualidade)
**Evolução dos preços da eletricidade para os agregados familiares**	↗ Aumento moderado (30% em comparação com a atualidade)	↗ Aumento moderado (30% em comparação com a atualidade)
	ESCOLHA	ESCOLHA

Table S1: General country statistics and key selection criteria for the case studies (in 2022), related to Star Method – Country selection.

General statistics	Denmark	Portugal	Poland	Germany
a) Population size in million	5.9	10.4	37.9	83.2
b) Geographical size (land area in km^2)	40,000	91,610	306,170	349,380
c) Share of rural population (in %)	12	33	40	22
d) GDP per capita in U$	68,000	24,600	18,000	51,200
e) Renewable electricity (annually in % of final demand)	65	58	16	45
f) Share of energy from renewable sources (% of gross final energy consumption)	31.6	34	16.1	19.3
g) Wind power capacity (MW)	6,260	5,120	6,300	62,190
h) Wind power capacity per capita (MW/person)	0.11	0.05	0.02	0.07

Table S2; Comparison of sample with population, related to Results and Method-Sampling

Table S1: Characteristics of sample versus population in selected demographic variables

Country	Demographic	Level	Sample (in%)	Population (in %)*
Denmark				
	Age	15-24	2.6	14.6
		25-29	3.2	8.2
		30-39	5.7	14.5
		40-49	8.9	13.8
		50-64	25.4	23.5
		65-74	32.4	19.9
		75-100	20.6	11.5
	Gender	Male	46.5	49.7
		Female	53.4	50.2
	Area	Urban	74.6	88
		Rural	25.4	12
Germany				
		15-24	6.4	11.7
		25-29	7.1	6.9
		30-39	14.0	15.3
		40-49	13.4	14.1
		50-64	33.6	26.2
		65-74	20.7	13.0
		75-100	4.8	12.8
	Gender	Male	49.2	49.3
		Female	50.7	50.6
	Area	Urban	64.8	88
		Rural	35.2	78
Poland				
		15-24	13.8	15.9
		25-29	9.7	6.3
		30-39	24.7	16.7
		40-49	19.5	17.3
		50-64	26.5	20.3
		65-74	5.6	13.2
		75-100	0.3	10.4
	Gender	Male	49.8	48,3
		Female	49.8	51.6
	Area	Urban	63.5	60
		Rural	36.5	40
Portugal				
		15-24	4.1	12.0
		25-29	8.1	6.0
		30-39	20.4	13.2
		40-49	26.7	17.0
		50-64	26.0	24.5
		65-74	13.9	14.0
		75-100	0.5	13.3
	Gender	Male	49.9	47.6
		Female	52.0	52.3
	Area	Urban	71.1	67
		Rural	28.9	33

*Total population age of 15 and above. Please note, due to non-conformity of population data regarding age structures, the first level start at 15+, however our sample only comprised 18+.

Sources: [1] Statistics Denmark 2022. Link: https://www.statbank.dk/20021; [2] Statistics Germany 2022. Link: https://www.destatis.de/; [3]Statistics Poland 2022. Link: https://stat.gov.pl; [4] Statistics Portugal 2022. Link: https://www.pordata.pt/en

Table S3: Diagnostic sample statistics, related to Method – Data analysis

Table S2: Sample statistics of the posterior distributions of all parameters in the base model. In addition to mean and standard deviation, this table shows the effective sample size (ESS) in the bulk and tail of these distributions, and the criteria measuring convergence of the four Markov chains we use (R-hat).

Parameter	Mean	sd	ESS (bulk)	ESS (tail)	R-hat
alpha[TECHNOLOGY:Open-field PV]	-0.14	0.07	3548	2719	1.00
alpha[TECHNOLOGY:Wind]	-0.49	0.09	4453	4254	1.00
alpha[LAND:1%]	0.02	0.08	3850	3072	1.00
alpha[LAND:2%]	0.04	0.06	4269	2757	1.00
alpha[LAND:4%]	-0.03	0.05	5451	3938	1.00
alpha[LAND:8%]	-0.12	0.07	5470	4866	1.00
alpha[TRANSMISSION:+0% .]	-0.06	0.08	5092	4074	1.00
alpha[TRANSMISSION:+25% .]	-0.07	0.06	4822	3013	1.00
alpha[TRANSMISSION:+50% .]	-0.15	0.09	4791	4155	1.00
alpha[TRANSMISSION:+75% .]	-0.20	0.09	5761	4147	1.00
alpha[SHARE_IMPORTS:10%]	-0.10	0.09	2961	1837	1.00
alpha[SHARE_IMPORTS:50%]	-0.41	0.15	2946	2584	1.00
alpha[SHARE_IMPORTS:90%]	-0.68	0.22	3764	4258	1.00
alpha[PRICES:+15%]	-0.25	0.06	5344	3567	1.00
alpha[PRICES:+30%]	-0.56	0.11	3586	3611	1.00
alpha[PRICES:+45%]	-0.98	0.17	4087	4326	1.00
alpha[PRICES:+60%]	-1.39	0.26	3521	3628	1.00
alpha[OWNERSHIP:Community]	-0.06	0.05	6859	4624	1.00
alpha[OWNERSHIP:Private]	-0.23	0.10	4329	3946	1.00
mu_left_intercept	0.09	0.04	9863	6252	1.00
sigma_country[TECHNOLOGY:Open-field PV]	0.10	0.10	1988	2681	1.00
sigma_country[TECHNOLOGY:Wind]	0.15	0.11	2342	2507	1.00
sigma_country[LAND:1%]	0.11	0.10	2304	3156	1.00
sigma_country[LAND:2%]	0.06	0.07	2897	2969	1.00
sigma_country[LAND:4%]	0.05	0.07	3828	4503	1.00
sigma_country[LAND:8%]	0.10	0.09	2567	3615	1.00
sigma_country[TRANSMISSION:+0% .]	0.11	0.10	2443	3095	1.00
sigma_country[TRANSMISSION:+25% .]	0.06	0.07	2868	3440	1.00
sigma_country[TRANSMISSION:+50% .]	0.14	0.11	2320	1964	1.00
sigma_country[TRANSMISSION:+75% .]	0.13	0.11	3066	3552	1.00
sigma_country[SHARE_IMPORTS:10%]	0.12	0.11	2247	2923	1.00
sigma_country[SHARE_IMPORTS:50%]	0.24	0.15	3624	3613	1.00
sigma_country[SHARE_IMPORTS:90%]	0.42	0.20	5708	5690	1.00
sigma_country[PRICES:+15%]	0.07	0.08	3134	3980	1.00
sigma_country[PRICES:+30%]	0.18	0.13	3106	2720	1.00
sigma_country[PRICES:+45%]	0.31	0.18	4915	5026	1.00
sigma_country[PRICES:+60%]	0.47	0.24	5449	5169	1.00
sigma_country[OWNERSHIP:Community]	0.05	0.06	4249	4200	1.00
sigma_country[OWNERSHIP:Private]	0.17	0.12	3969	4921	1.00
sigma_respondent[TECHNOLOGY:Open-field PV]	0.59	0.05	1420	2629	1.00
sigma_respondent[TECHNOLOGY:Wind]	1.06	0.04	1568	3367	1.00

Parameter	Mean	sd	ESS (bulk)	ESS (tail)	R-hat
sigma_respondent[LAND:1%]	0.12	0.09	553	1166	1.00
sigma_respondent[LAND:2%]	0.20	0.12	378	1202	1.01
sigma_respondent[LAND:4%]	0.15	0.10	517	909	1.01
sigma_respondent[LAND:8%]	0.22	0.12	334	1145	1.01
sigma_respondent[TRANSMISSION:+0% .]	0.11	0.08	639	1195	1.00
sigma_respondent[TRANSMISSION:+25% .]	0.10	0.07	657	1314	1.00
sigma_respondent[TRANSMISSION:+50% .]	0.22	0.12	396	1061	1.01
sigma_respondent[TRANSMISSION:+75% .]	0.50	0.08	677	833	1.00
sigma_respondent[SHARE_IMPORTS:10%]	0.25	0.11	317	404	1.01
sigma_respondent[SHARE_IMPORTS:50%]	0.12	0.08	496	1105	1.01
sigma_respondent[SHARE_IMPORTS:90%]	0.83	0.05	1583	3230	1.00
sigma_respondent[PRICES:+15%]	0.15	0.10	374	945	1.01
sigma_respondent[PRICES:+30%]	0.07	0.05	1523	2231	1.00
sigma_respondent[PRICES:+45%]	0.66	0.06	1347	2724	1.00
sigma_respondent[PRICES:+60%]	1.39	0.06	1532	3334	1.00
sigma_respondent[OWNERSHIP:Community]	0.11	0.08	452	967	1.02
sigma_respondent[OWNERSHIP:Private]	0.59	0.05	912	1980	1.01
sigma_left_intercept	0.10	0.07	399	781	1.02

Note S1: Country case selection, related to Method - Country selection

The four country cases where selected to reflect diversity across geographical, economic and (energy) historical characteristics and the progress of their national energy transitions. Germany was chosen as the largest EU country with a highly urbanised population and living standard and well progressed energy transition. In contrast, Denmark in the north and Portugal in the south, have even further progressed energy transitions but are much smaller in geographical and population size. Poland was selected as an eastern European country, large both in size and population but with a significantly lower share in renewable energy. In addition, Portugal and Poland have a lower level of economic development and living standards in very different geographical locations and cultural-historic settings.
